# Three‐Dimensional Reconstruction of Cephalopod Beaks: A 3D Modeling Workflow Combining Prism‐Assisted Photogrammetry and Micro‐CT Validation

**DOI:** 10.1002/ece3.73992

**Published:** 2026-07-12

**Authors:** Lele Xu, Yongqin Li, Jiechun Chen, Dongmei Huang, Yao Liu, Liyun Wang, Bilin Liu

**Affiliations:** ^1^ Life Science and Technology School Lingnan Normal University Zhanjiang China; ^2^ Western Guangdong Provincial Engineering Technology Research Center of Seafood Resource Sustainable Utilization Lingnan Normal University Zhanjiang China; ^3^ School of Electronic and Electrical Engineering Lingnan Normal University Zhanjiang China; ^4^ College of Marine Sciences, Shanghai Ocean University Shanghai P. R. China

**Keywords:** 3D model, 3D reconstruction, cephalopod beak, micro‐CT validation, photogrammetry, prism reflection

## Abstract

Cephalopod beaks contain rich ecological information, but their small size and sensitivity to dehydration make accurate three‐dimensional (3D) reconstruction challenging. We describe a validated workflow that integrates prism‐assisted photogrammetry, digital sculpting, and micro‐CT validation to produce accurate 3D models of cephalopod beaks. Using the upper beak of *Sepia pharaonis* as a case study, a prism‐reflection imaging system simultaneously captured five isometric views in a single photograph, reducing acquisition time to under 1 min. Hierarchical cluster analysis demonstrated nearly 100% species‐level discrimination between *S. pharaonis* and *Sepioteuthis lessoniana* , with digital models, 3D‐printed replicas, and original specimens converging at fine resolution. Digital model measurements deviated from micro‐CT reference values by < 2% for linear dimensions and < 3° for angular measurements, with coefficients of variation of 3.5%–5.7% across morphological parameters. This cost‐effective and portable approach removes the technical bottleneck limiting large‐scale beak‐based ecological analyses, enabling applications in population structure inference, diet reconstruction, and demographic modeling.

## Graphical abstract

This study presents a novel workflow for three‐dimensional reconstruction of small, dehydration‐sensitive biological specimens, demonstrated through cephalopod beak modeling. The method combines prism‐assisted photogrammetry—which captures five isometric views in under 60 s using 45° prism reflections—with digital sculpting in ZBrush and micro‐CT validation. Results show high accuracy (< 2% linear error, < 3° angular error) and low coefficient of variation (3.5%–5.7%), with successful species‐level discrimination between *Sepia pharaonis* and *Sepioteuthis lessoniana* , offering a cost‐effective alternative to micro‐CT for ecological and evolutionary research.

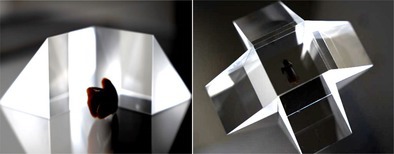

## 1 Introduction

Cephalopod beaks are the primary feeding organs of octopuses, cuttlefish, and squid. They contain rich ecological and biological information, making them invaluable for studies of age identification, population structure, growth estimation, migration patterns, and habitat reconstruction (Schwarz et al. 2019; Liu et al. 2020; Caldwell et al. 2015). As one of the few hard tissue structures in these soft‐bodied organisms, beaks offer practical advantages: easy extraction, stable morphological structure, and resistance to corrosion (Zhang et al. 2021).

From a population ecology perspective, cephalopod beak morphology carries robust ecological signals. Beak shape and size correlate with ontogenetic stage, body size, and dietary niche, and have been used to infer demographic parameters such as age at maturity and growth rates (Perales‐Raya et al. 2010; Liu et al. 2020). In trophic ecology, predator–prey interactions are frequently reconstructed from beak remains found in stomach contents, and quantitative 3D morphological data could improve the taxonomic resolution of such diet studies. However, most large‐scale beak‐based ecological studies still rely on simplified two‐dimensional measurements or manual caliper data, which may obscure functionally important shape variation. A validated, low‐cost 3D reconstruction pipeline is therefore needed to scale beak‐based ecological analyses to the population and community levels.

Accurate 3D reconstruction of cephalopod beaks remains technically demanding. Beaks are typically small (2–5 cm), dehydrate and deform rapidly when exposed to air, exhibit complex curved geometries, and display variable pigmentation ranging from black at the rostral tip to transparent at the edges (Miserez et al. 2008). Traditional methods such as manual measurement or two‐dimensional imaging fail to capture the full 3D morphology required for functional and comparative analyses.

Recent advances in 3D imaging have provided two primary approaches: micro‐computed tomography (micro‐CT) and photogrammetry. Micro‐CT offers high precision but requires expensive equipment, specialized facilities, and lengthy scanning times (Roscian et al. 2021). Photogrammetry, including the underwater approach demonstrated by Roscian et al. (2021, 2022), provides a more accessible alternative but typically requires complex setups and extended imaging times that may exceed the critical window before specimen deformation occurs.

Accurate 3D models of biological specimens have received increasing attention in biological research (Corral‐Acero et al. 2020; Peirlinck et al. 2021). Such models enable non‐destructive analysis, quantitative morphological comparisons, and broader dissemination through 3D printing. However, producing accurate 3D models of small, delicate specimens requires methods that balance precision, speed, and accessibility.

Here, we describe a workflow that combines prism‐assisted photogrammetry, digital sculpting software, and micro‐CT validation. Our method uses the principle of light reflection through 45° prisms to simultaneously capture five isometric views in a single photograph, reducing acquisition time to under 1 min—a critical advantage for dehydration‐sensitive specimens. This approach represents the first systematic application of prism reflection principles to small biological sample imaging, establishing a complete 3D modeling workflow for cephalopod beak morphology.

The present study is framed as a pilot methodological investigation that establishes the accuracy and feasibility of this low‐cost 3D reconstruction pipeline. By validating the method against micro‐CT and demonstrating its capacity for species discrimination via hierarchical clustering, we provide a foundation for downstream ecological applications at the population and community levels.

Our objectives were to: (1) develop and test a rapid, cost‐effective method for 3D reconstruction of small, delicate biological specimens, using the upper beak of *Sepia pharaonis* Ehrenberg, 1831, as a case study; (2) validate the accuracy of resulting 3D models against micro‐CT reference measurements; and (3) demonstrate the method's applicability to cephalopod beak morphology and species discrimination. This workflow is faster and less costly than existing alternatives, with potential applications in ecological and evolutionary research.

## 2 Material and Methods

### 2.1 Specimen Collection and Preparation

A Pharaoh cuttlefish ( *Sepia pharaonis* , mantle length ~25 cm, weight ~2 kg) was obtained from a local fishery market in Zhanjiang, Guangdong Province, China. The upper and lower beaks were extracted from the buccal mass using fine tweezers and immediately preserved in 70% ethanol in seawater, following the protocol of Perales‐Raya et al. (2010). Additional beaks from the bigfin reef squid ( *Sepioteuthis lessoniana* Lesson, 1830) were obtained for comparative validation.

**FIGURE 1:**
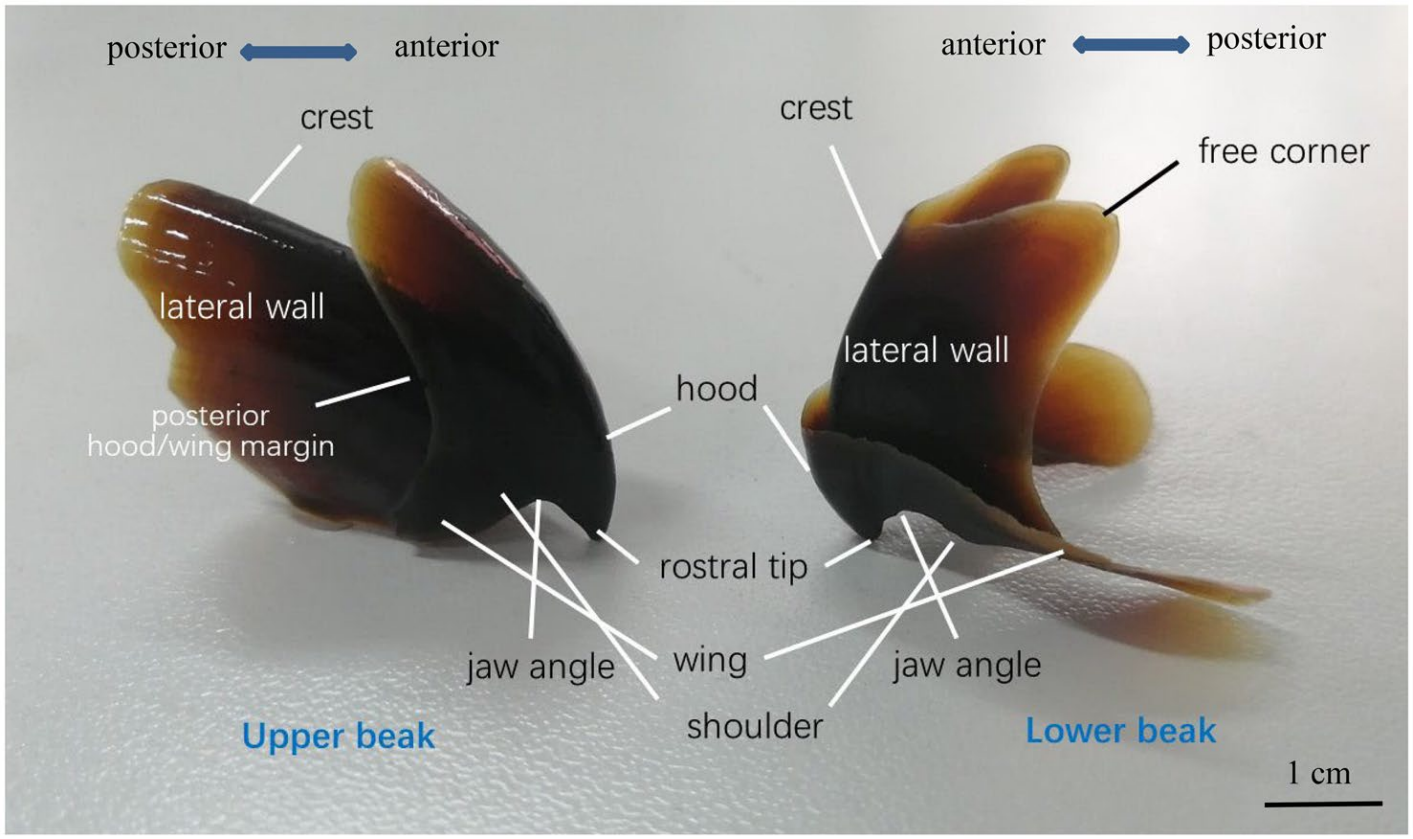
Anatomy of cuttlefish *Sepia pharaonis* beak with terminology proposed by Clarke (1986).

**FIGURE 2:**
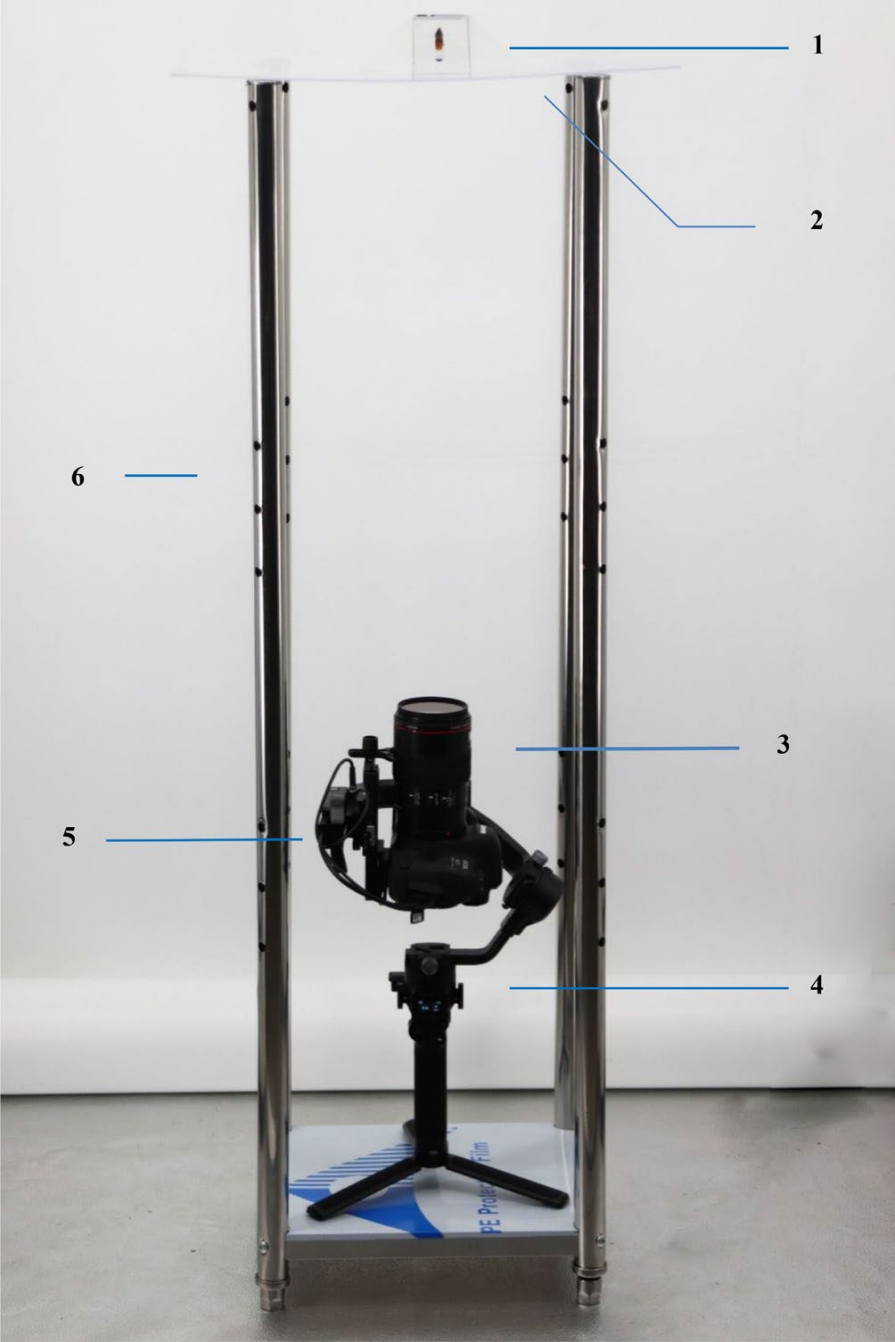
The photogrammetry apparatus. It is placed in a 200 × 120 × 100 cm portrait studio to obtain sufficient incident light. (1) Sample with prisms surrounded. (2) The acrylic plate. (3) Canon EF 100 mm f/2.8 L Macro IS USM Lens. (4) Camera gimbal. (5) Image transmitter system. (6) White reflective fabric.

**FIGURE 3:**
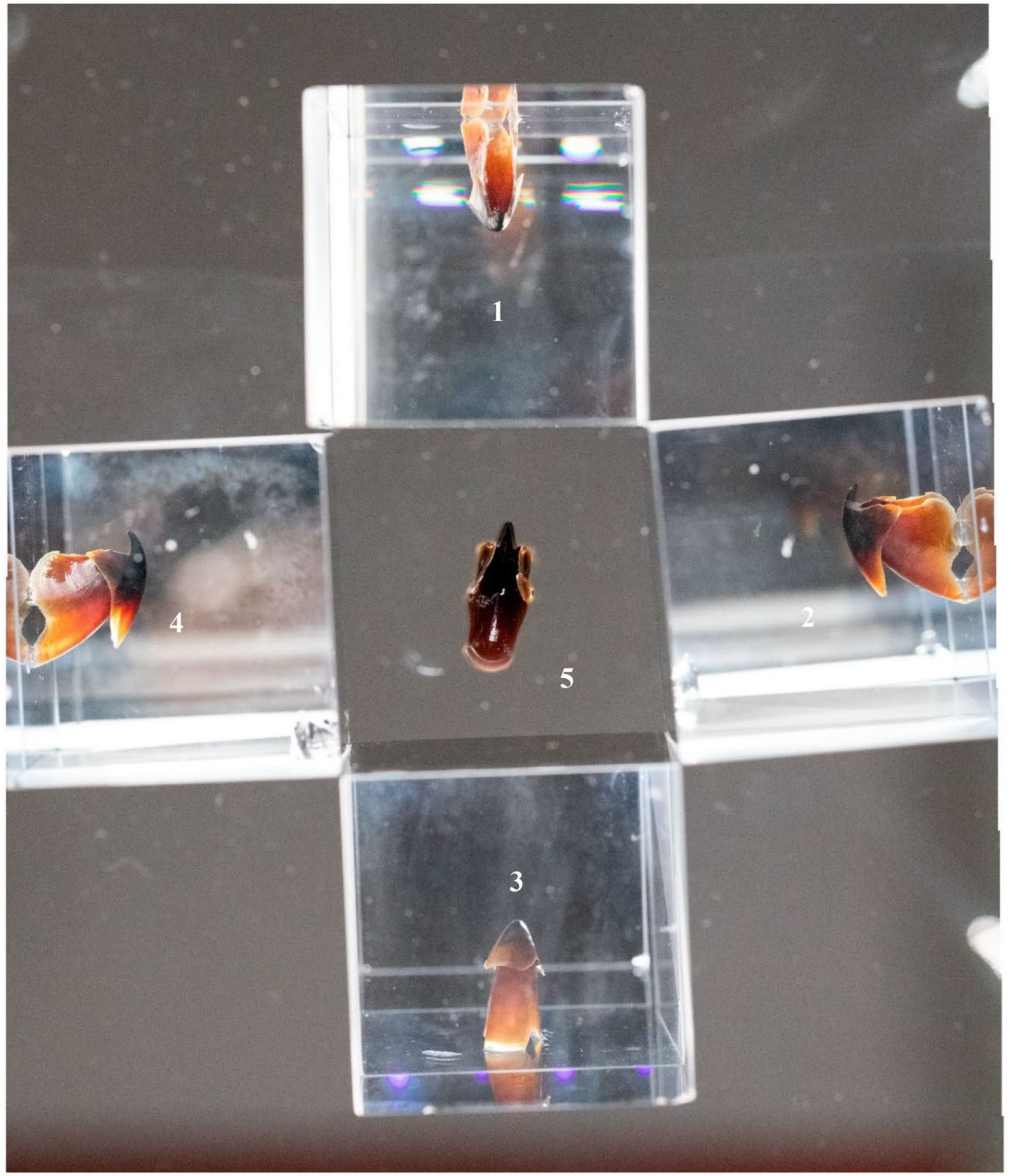
The images of the five‐view of the beak that took from a camera placed in the bottom of the prism, that is, front view (1), left view (2), rear view (3), right view (4), from clockwise up to down, and bottom view (5) in the center.

Anatomical terminology follows Clarke (1986), with the beak divided into upper and lower components, each comprising the rostra, rostral tip, hood, jaw angle, wings, crest, and lateral walls (Figure 1). The pigmentation gradient from black (rostral tip) to transparent (peripheral edges) corresponds to hardness gradients, with the rostral tip being the hardest and most heavily pigmented region. For brevity, the upper beak is used hereafter as the exemplar to illustrate the modeling workflow (Figures 2 and 3).

### 2.2 Prism‐Assisted Photogrammetry System

#### 2.2.1 Apparatus Design

**FIGURE 4:**
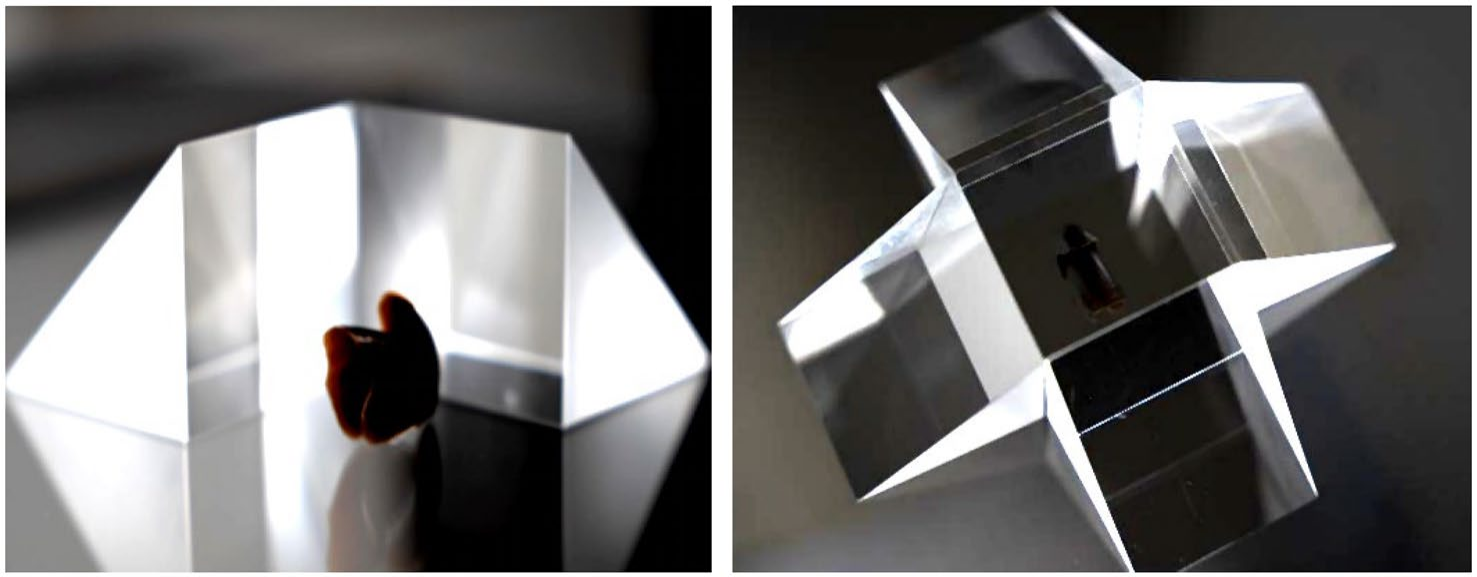
The placement (layout) of prisms, which formed a wall that surrounded the upper beak in the center of them.

A novel photogrammetry system was designed based on the principle of light reflection through 45° prisms (Figure 4). The apparatus consisted of: (i) a stainless‐steel support frame (120 cm height × 30 cm width) with a transparent acrylic platform; (ii) four triangular prisms (50 × 50 × 50 mm, K9 optical glass, *n* = 1.5163) arranged in a square configuration around the specimen; (iii) a camera gimbal (DJI RSC 2) mounted beneath the platform, holding a Canon EOS 6D Mark II with an EF 100 mm f/2.8 L Macro IS USM lens; (iv) a portrait studio enclosure (200 × 120 × 100 cm) with multiple LED light sources providing homogeneous illumination (750–1500 lx); and (v) a polarizing filter attached to the lens to minimize reflections from the acrylic platform.

#### 2.2.2 Image Acquisition Protocol

The prism configuration exploited total internal reflection to simultaneously capture four side views (front, left, rear, and right) on the prism faces, plus a bottom view through the central aperture (Figure 4). Because the four side views reflected on the prism faces and the bottom view through the central aperture occupy different focal planes, a single photograph cannot render all five views in sharp focus simultaneously. To overcome this limitation, two bracketed photographs were captured for each specimen using identical camera settings (f/11, 1/125 s, ISO 100): the first focused on the prism faces to capture the side views, and the second focused on the central aperture to capture the bottom view.

These two images were imported into Adobe Photoshop 2023 as separate layers. The Auto‐Align Layers function (Edit > Auto‐Align Layers, Projection: Auto) was first applied to correct for minor optical shifts between frames. The Auto‐Blend Layers command (Edit > Auto‐Blend Layers, Blend Method: Stack Images, with Seamless Tones and Colors enabled) was then used to merge the sharply focused regions from each layer into a single composite image. This focus‐stacking procedure yielded an extended depth‐of‐field photograph in which all five views (front, left, rear, right, and bottom) were rendered in sharp focus, preserving fine surface details such as the rostral tip texture (~100 μm features) and the transparent peripheral edges essential for accurate downstream contour extraction. The total acquisition time was under 60 s per specimen—substantially faster than underwater photogrammetry protocols (Roscian et al. 2021) and well within the critical window before dehydration‐induced deformation (> 10 min under subtropical conditions).

### 2.3 Image Processing and Reference Library Construction

#### 2.3.1 Contour Extraction

**FIGURE 5:**
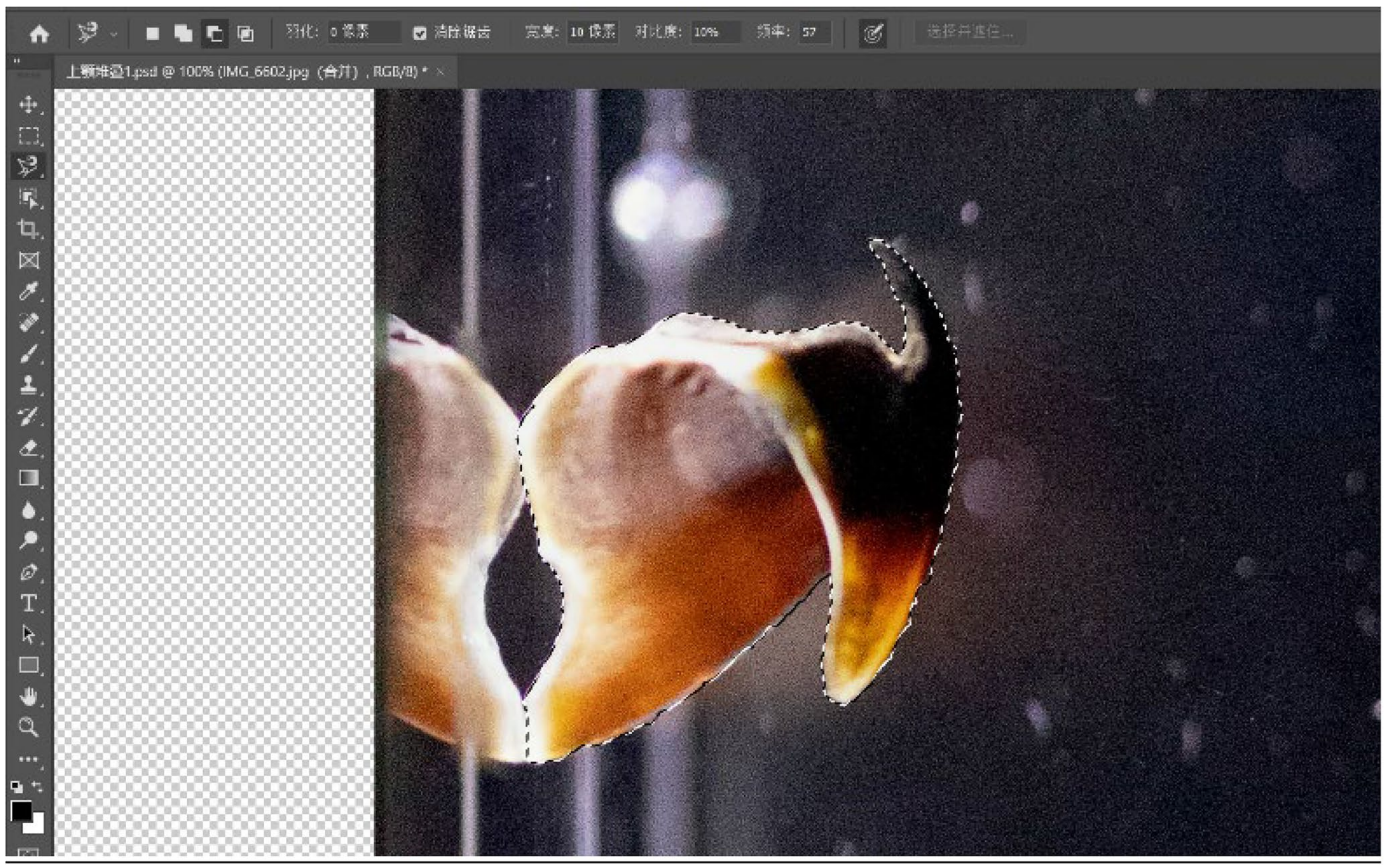
The extraction of the contour of the beak. Dashed line shows the selection area of the contour that has been established.

Raw images were processed in Adobe Photoshop CC following a standardized protocol (Figure 5):

1. Layer duplication: The background layer was duplicated to preserve the original image data.
2. Path creation: The magnetic lasso tool and pen tool were combined to delineate the beak contour, creating a closed path that was converted to a selection.
3. Edge refinement: The quick selection tool, with manual adjustments, was used to refine selection boundaries.
4. Channel masking: Individual RGB channels were evaluated, and the channel with the highest contrast was duplicated. The brush tool was used to paint beak regions white and the background black, generating a high‐contrast mask.
5. Color correction: Hue/saturation adjustments were applied to optimize color fidelity relative to the physical specimen.
6. Export: The isolated beak image was saved as a maximum‐quality JPEG.

#### 2.3.2 Isometric Reference Library

**FIGURE 6:**
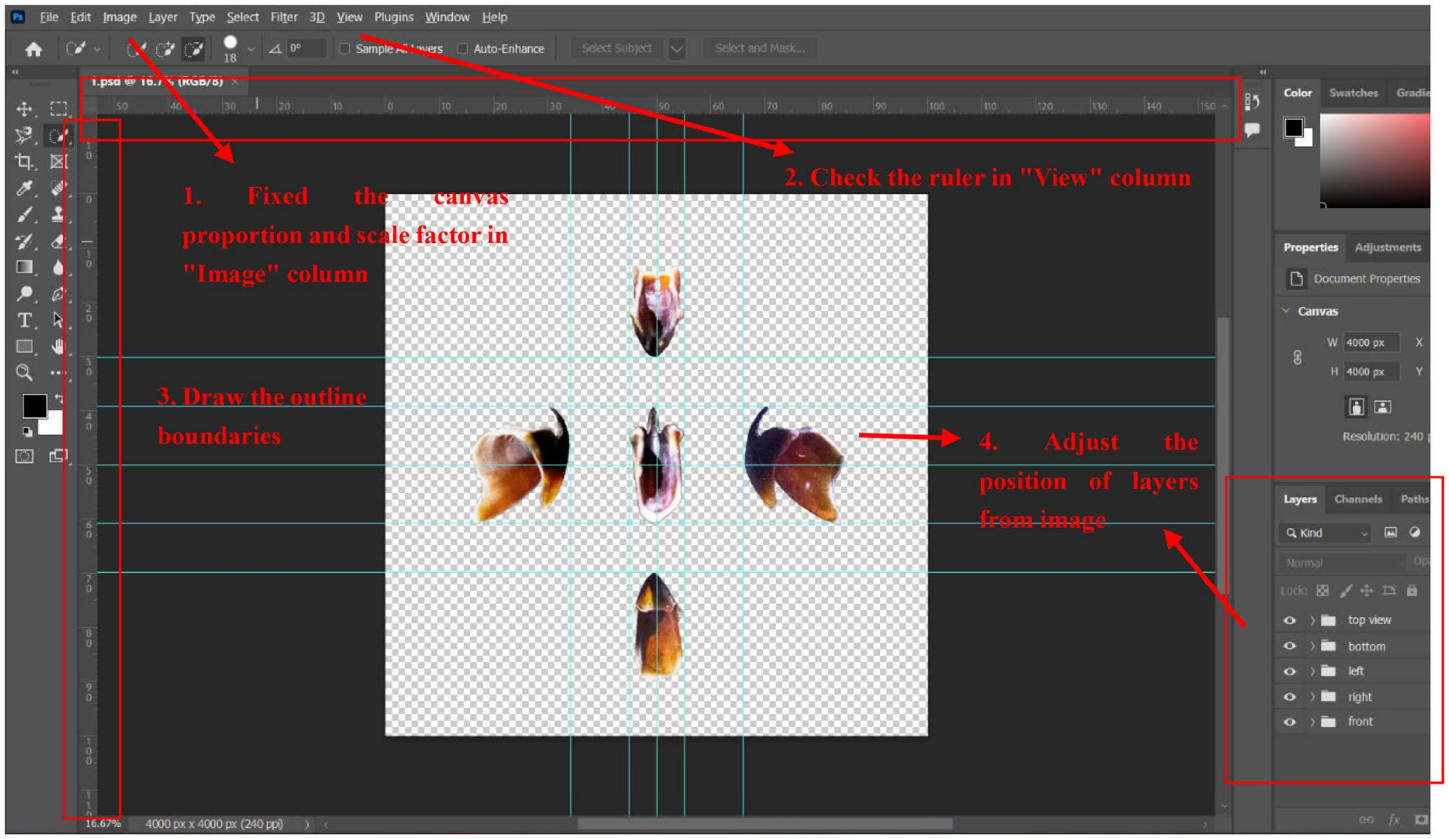
Set up rulers and reference lines in PS to create the isometric images of the beak from different orientations.

**FIGURE 7:**
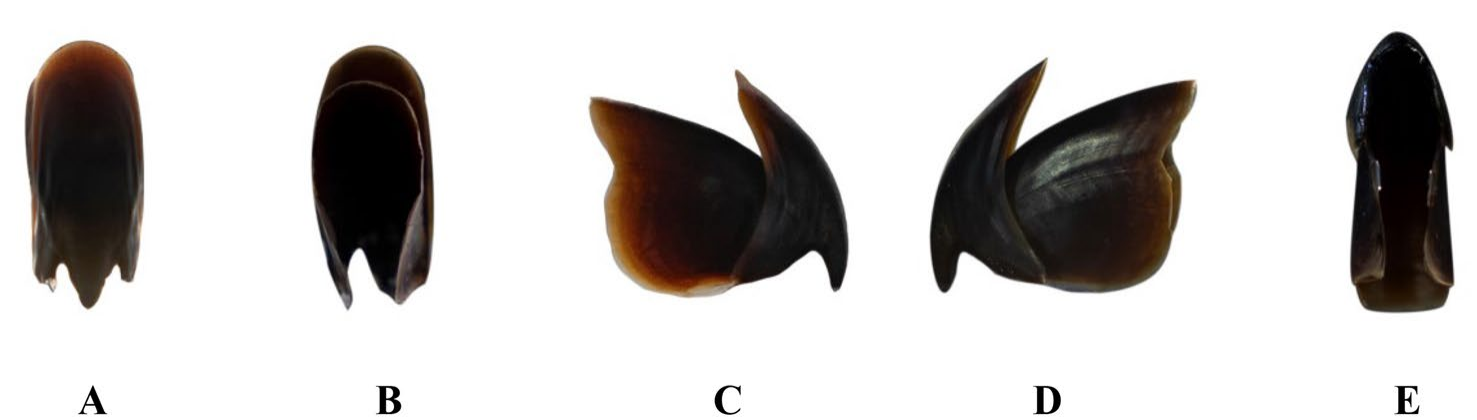
The diagram of isometric five‐angles of upper beak. A, B, C, D, and E are the front view, rear view, left view, right view, and bottom view respectively.

Processed images were assembled into an isometric reference library as follows:

1. Canvas standardization: Images were imported into 4000 × 4000 pixel canvases with locked aspect ratios.
2. Scale calibration: Rulers and guidelines were aligned to the beak contours, with the canvas centerline (50%) aligned to the center of the bottom view.
3. View extraction: Each of the five views (front, rear, left, right, and bottom) was cropped to 1200 × 1200 pixels using standardized wireframes.
4. Array assembly: Views were arranged in a standardized layout for 3D modeling reference (Figures 6 and 7).

### 2.4 3D Digital Reconstruction

#### 2.4.1 Software and Hardware

Digital sculpting was performed in ZBrush 2020 (Pixologic, USA) on a workstation equipped with an Intel Core i9‐10900K processor, 64 GB RAM, and an NVIDIA RTX 3080 graphics card. Micro‐CT validation was conducted using a Zeiss Metrotom‐1 (160 kV X‐ray source, 165 × 140 mm field of view, 5 μm voxel resolution).

#### 2.4.2 Base Mesh Creation

**FIGURE 8:**
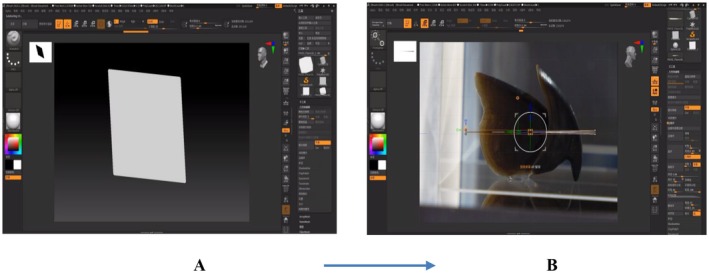
The prototype of the 3D model and reference images that have been imported for modeling in ZBrush software.

A primitive plane was converted to PolyMesh3D and iteratively subdivided to generate a high‐resolution base mesh. Using masking and the Split Unmasked Points function, the mesh was roughly shaped to match half of the upper beak morphology, leveraging bilateral symmetry (Figure 8A).

#### 2.4.3 Reference‐Guided Sculpting

The isometric reference library was imported into ZBrush using the Ground Grid system:

1. Ground grid activation: The X, Y, and Z reference planes were activated.
2. Image projection: Front/back, up/down, and left/right views were mapped to the corresponding Z, Y, and X planes, respectively.
3. Transparency optimization: Fill Mode was set to 3 for reference clarity, and Edge Opacity Coefficient was set to 1 for model edge visibility.
4. Back Point activation: Reference images remained visible behind the model during rotation.
5. Projection line: Enabled accurate cursor‐to‐reference mapping (Figure 8B).

#### 2.4.4 Detail Sculpting and Thickness Extraction

The Panel Loop function (Edge Rings = 1, Thickness = 0.08, Polish = 50) was used to extract wall thickness from the planar mesh. Large‐scale shape adjustments were performed using the FFD (Free Form Deformation) deformer, while the Move, Clay, and Standard brushes were used to refine surface details. Symmetry sculpting (X‐axis mirror) ensured bilateral consistency. The Smooth brush was applied to refine edges and transitions.

**FIGURE 9:**
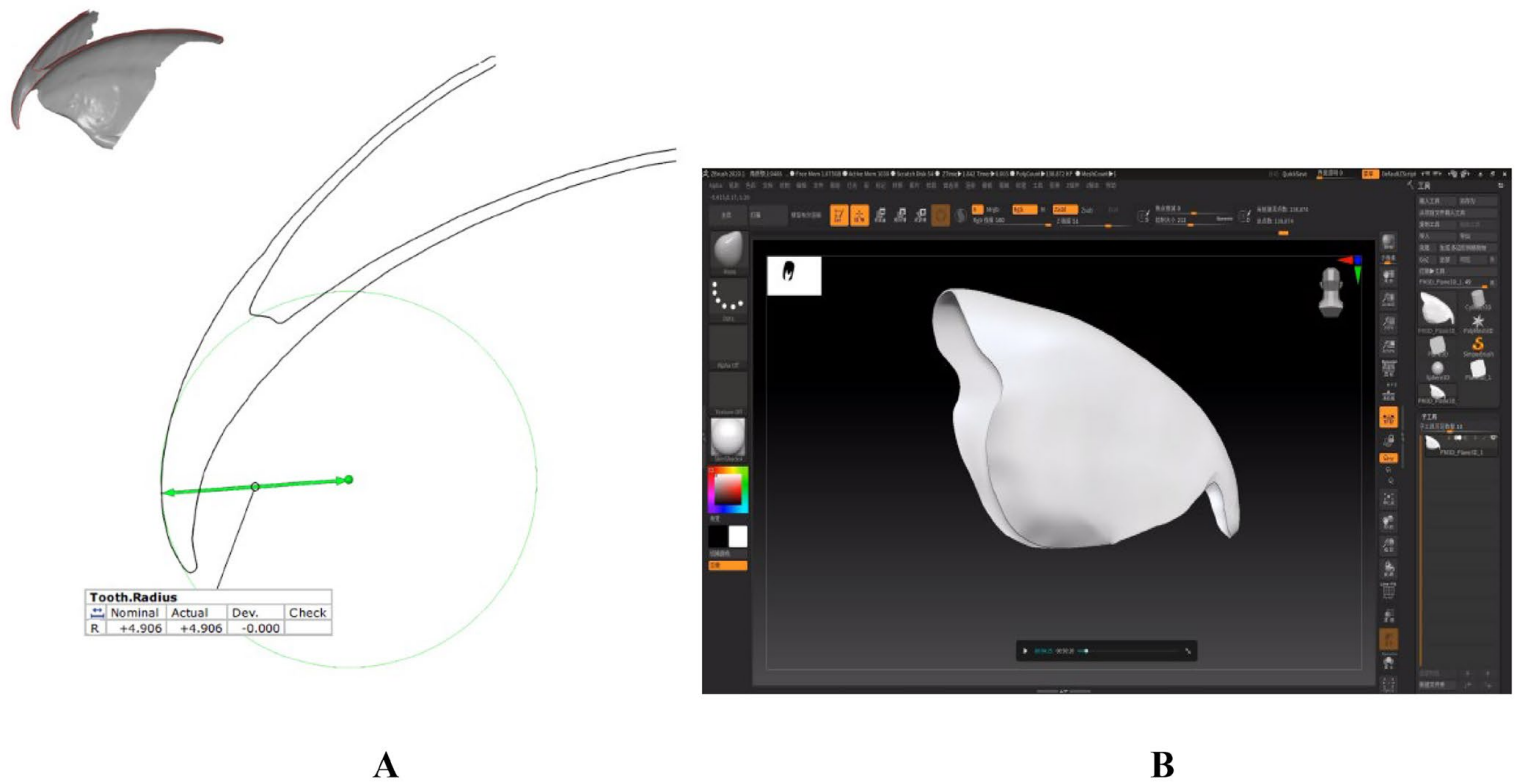
Using micro‐CT to assist modeling (A) and sculpt the model in detail in ZBrush software (B).

For operators unfamiliar with beak morphology, micro‐CT scans provided auxiliary guidance during initial model development (Figure 9A).

#### 2.4.5 Retopology and Material Assignment

Completed models underwent retopology using ZRemesher to generate clean edge loops suitable for measurement and 3D printing. Models were exported to Autodesk Maya 2025 for material assignment using the Standard Surface shader: base color was set to a dark brown‐to‐black gradient matching natural pigmentation; specularity was set to low‐to‐medium values for the chitinous surface; subsurface scattering was enabled for transparent edge regions; and sheen was added to simulate edge transparency effects (Figure 9B).

### 2.5 3D Printing and Physical Validation

#### 2.5.1 Print Preparation

STL files were processed in Materialize Magics for mesh repair and support generation. Printing parameters were as follows: UnionTech RSPro 600 stereolithography printer; photosensitive resin 9400 (white opaque, 0.10 mm precision); layer height 0.05 mm; minimum wall thickness 2.0 mm for structural integrity; and light supports with manual optimization for delicate features.

#### 2.5.2 Post‐Processing

Printed models were washed in 99% isopropanol for 5 min, cured under UV light (405 nm, 60 s per side), and support structures were carefully removed. The final dimensions of the printed models were: lower beak 31.069 × 27.454 × 32.200 mm; upper beak 42.193 × 27.218 × 30.500 mm.

### 2.6 Morphological Validation

#### 2.6.1 Micro‐CT Reference Measurements

Specimens were scanned using the Zeiss Metrotom‐1 at 5 μm resolution. Reconstructed volumes were segmented in VG Studio MAX 3.4, and 3D surface models were extracted for comparison.

Micro‐CT data were divided into two independent sets: a training dataset (*n* = 5) used exclusively for operator training in beak morphology and a validation dataset (*n* = 15: 8 *Sepia pharaonis* , 7 *Sepioteuthis lessoniana* ) for gold‐standard validation. Training and validation cohorts did not overlap, eliminating circular validation bias.

#### 2.6.2 Cluster Analysis

Morphological measurements (hood length, rostral length, crest height, jaw angle, lateral wall width) were extracted from digital models, printed replicas, and original specimens. Original specimens were measured with a vernier caliper (precision: 0.01 mm).

Hierarchical cluster analysis (Ward's method, Euclidean distance) was performed using SPSS software to evaluate morphological fidelity and species discrimination.

#### 2.6.3 Method Reliability Assessment

Three representative specimens (2 *Sepia pharaonis* and 1 *Sepioteuthis lessoniana* upper beaks) were independently imaged and reconstructed five times by the same operator to evaluate repeatability. Five morphological metrics (hood length, crest length, rostrum length, rostrum width, and wing length) were measured from each model.

## 3 Results

### 3.1 Photogrammetry System Performance

The prism‐assisted photogrammetry system captured five isometric views in under 60 s, compared to 15–30 min for underwater photogrammetry protocols (Roscian et al. 2021). The acrylic platform and polarizing filter eliminated specular reflections, and homogeneous LED illumination provided consistent, shadow‐free lighting.

Focus‐stacked composite images retained an extended depth of field, capturing fine details of the rostral tip (~100 μm features) and transparent edges.

### 3.2 3D Model Accuracy

#### 3.2.1 Visual Comparison

**FIGURE 10:**
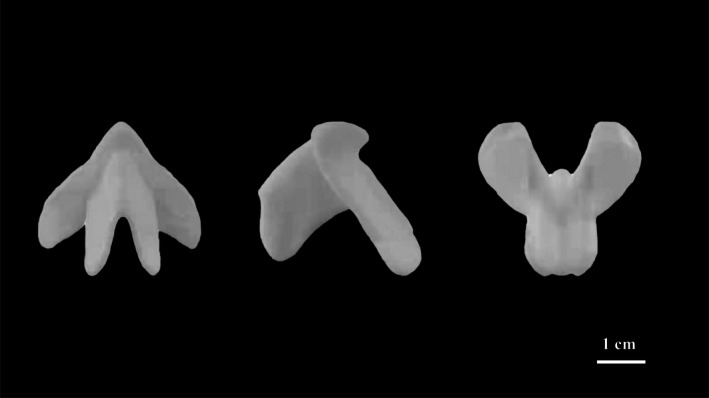
The three views of the solid model of the lower beak in the real world. The model material used was resin 9400.

Digital models rendered in ZBrush showed no visible morphological differences from micro‐CT reconstructions. Key anatomical features—rostral tip curvature, hood profile, crest height, lateral wall splay, and jaw angle—were accurately reproduced. The digital sculpting approach captured subtle surface textures and pigmentation gradients that photogrammetry alone could not resolve (Figure 10).

#### 3.2.2 Quantitative Validation

**FIGURE 11:**
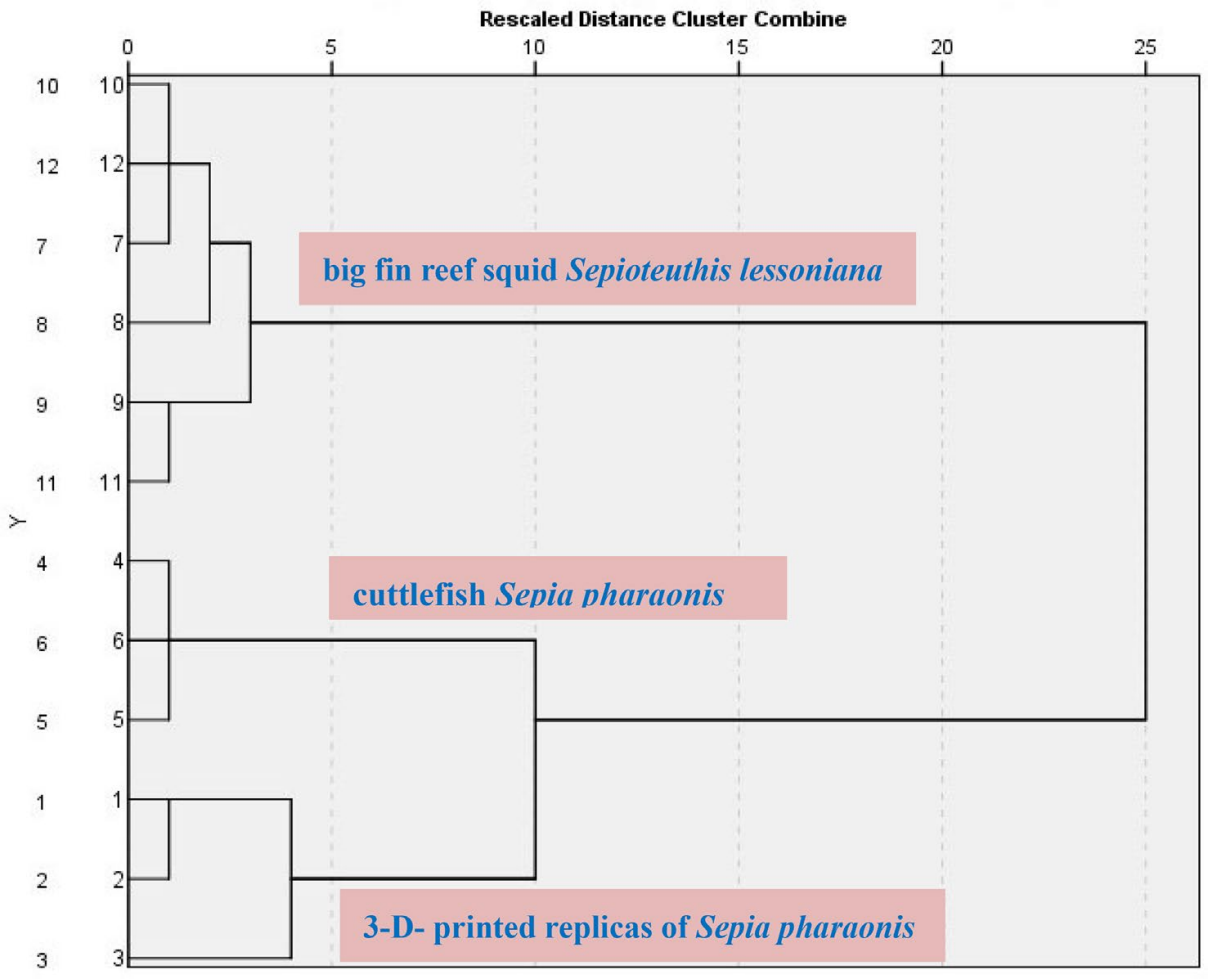
The result of hierarchical cluster analysis of beaks. Number 4–6 are the beaks of cuttlefish *Sepia pharaonis* ; Number 1–3 represent the 3‐D printing model from prototype of number 4–6 Number. Number 7–12 are the beaks from big fin reef squid, *Sepioteuthis lessoniana* .

Hierarchical cluster analysis of morphological measurements (*n* = 12: 6 *Sepia pharaonis* , 6 *Sepioteuthis lessoniana* , all purchased from local markets) demonstrated:

- Species‐level discrimination: At Euclidean distance = 15, specimens clustered into two distinct groups corresponding to species (100% accuracy).
- Method convergence: At Euclidean distance = 5, digital models, printed replicas, and original specimens of the same species clustered together, indicating morphological fidelity across the digital‐to‐physical workflow (Figure 11).
- Measurement precision: Digital model measurements deviated from micro‐CT reference values by < 2% for linear dimensions and < 3° for angular measurements.
- Method repeatability: Coefficient of variation (CV) values ranged from 3.5% to 5.7% across all morphological parameters, confirming procedural reliability (Table 1).

**TABLE 1:** The average coefficient of variation (CV) of beak morphological parameters.

Morphological parameters	*Sepia pharaonis*		*Sepioteuthis lessoniana*	
	Average/mm	CV/%	Average/mm	CV/%
Hood length	12.25	4.8	15.56	3.5
Wing length	13.31	5.2	6.95	5.7
Rostrum length	3.76	3.6	4.50	4.8
Rostrum width	3.09	3.9	4.01	4.5
Crest length	16.12	4.1	21.21	4.6

### 3.3 Physical Model Quality

**FIGURE 12:**
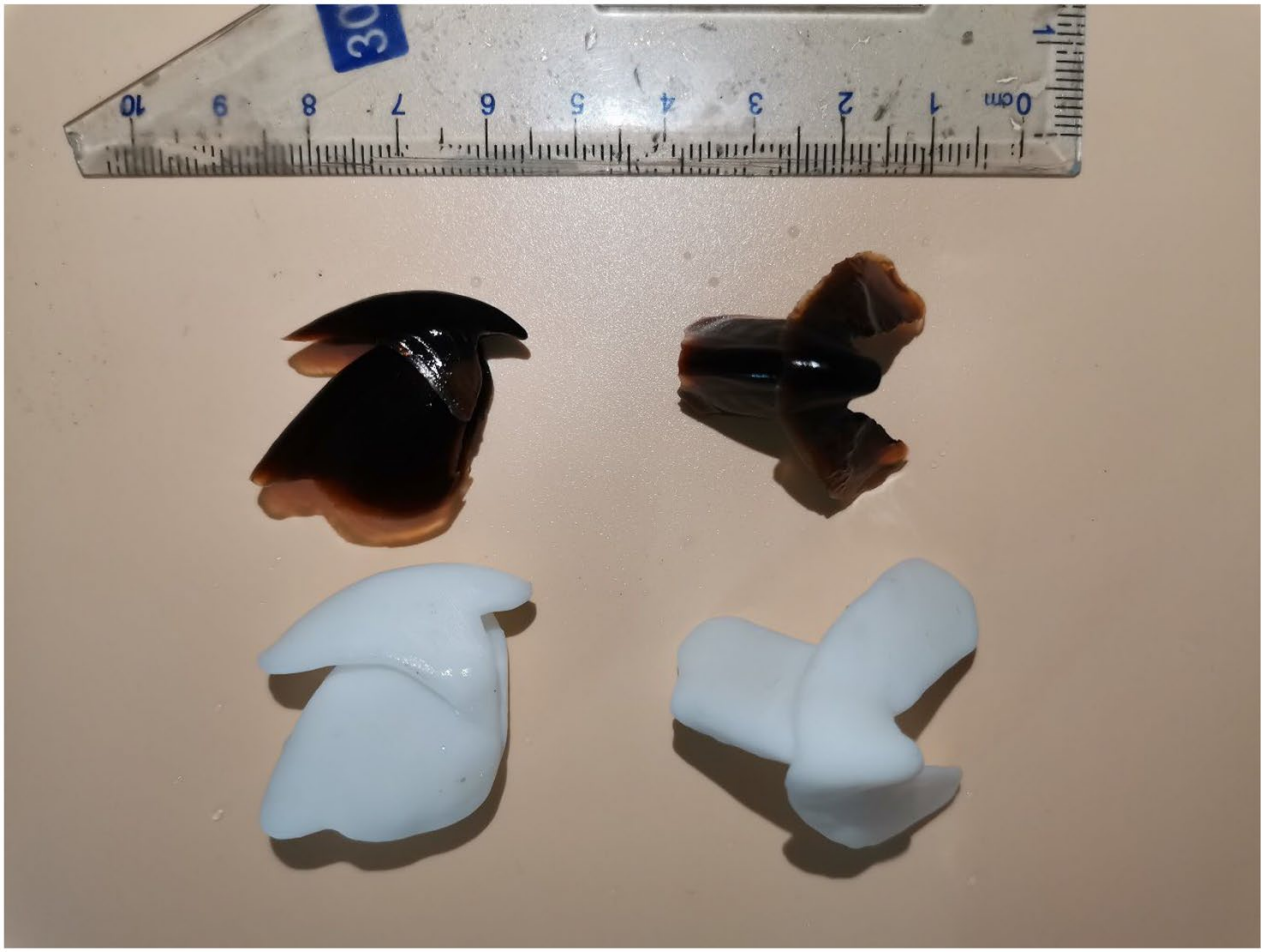
Comparison of the real cuttlefish beak (up) and its cloned plastic model (down).

3D‐printed replicas reproduced the digital models with dimensional accuracy within ±0.15 mm (0.5% relative error). The white resin material required post‐processing painting to match natural pigmentation, but structural fidelity was excellent (Figure 12). The 2 mm minimum wall thickness requirement resulted in slightly thicker edges than the original (magnification factor ~20×), though overall proportions and morphological relationships were preserved.

## 4 Discussion

### 4.1 Methodological Innovation: Prism‐Assisted Photogrammetry

This study represents the first systematic application of prism reflection principles to small biological specimen imaging. While Corti et al. (1996) and He et al. (2020) previously used prisms for multi‐view imaging of rodent mandibles and cephalopod beaks, their systems required complex multi‐camera setups or enclosed imaging boxes. Our generalized approach replaces these with standard photography equipment (DSLR camera, gimbal stabilizer) and an open prism configuration, substantially improving accessibility and reducing cost.

The key innovation lies in exploiting total internal reflection in 45° prisms to create multiple virtual camera viewpoints from a single physical camera position. This configuration offers several advantages for biological imaging:

1. Speed: Sub‐minute acquisition prevents dehydration‐induced deformation in sensitive specimens.
2. Scalability: The 5 × 5 × 5 cm imaging volume can be adjusted by changing prism size for specimens ranging from insects to small vertebrates.
3. Standardization: Fixed prism geometry ensures consistent isometric views across specimens and operators.
4. Cost: The complete apparatus costs < $100 USD (excluding camera and commercial software; costs could be reduced further using open‐source alternatives such as Blender), compared to > $50,000 for micro‐CT systems or $5000–10,000 for underwater photogrammetry setups.

### 4.2 3D Model Accuracy Assessment

Our hierarchical cluster analysis shows that software‐based digital sculpting, when guided by accurate reference images, can achieve accuracy comparable to reverse‐engineering approaches (micro‐CT, photogrammetry + Geomagic) at lower cost. This accuracy depends on three factors:

- Accurate reference images: Prism‐assisted photogrammetry provides sub‐millimeter accuracy in 2D projections.
- Operator expertise: Digital sculpting relies on human pattern recognition to interpret ambiguous regions (e.g., transparent edges).
- Validation procedure: Micro‐CT data were used exclusively for operator training and were not included in final model validation. Validation was performed on 15 independent samples (8 *Sepia pharaonis* , 7 *Sepioteuthis lessoniana* ) excluded from the training dataset, ensuring complete separation between cohorts.

The < 2% linear measurement error and < 3° angular error are comparable to or exceed reported accuracies for 3D‐printed medical replicas (Sokolowski et al. 2019; Johnson et al. 2021), validating the method's suitability for rigorous morphological research. Volumetric validation—direct comparison of beak volumes between micro‐CT voxel reconstructions and photogrammetry‐derived closed‐mesh models—was not performed in this study but represents a logical direction for future methodological refinement.

### 4.3 Comparison With Existing Methods

Our method is suited to researchers requiring accurate external morphology models with limited equipment. The learning curve for ZBrush (~2 months for proficiency) is the main barrier to adoption, offset by the software's widespread availability and extensive educational resources (Table 2).

**TABLE 2:** Comparison of 3D reconstruction methods for small biological specimens.

Items	Software 3‐D modeling & printing (this Study)	Underwater photogrammetry	Micro‐CT scanning
Equipment cost	< $1000 (camera + software)	$5000–10,000	$50,000–500,000
Image acquisition time	< 1 min	15–30 min	2–8 h
Processing time	4–6 h	2–4 h	1–2 h
Portability	High (portable studio)	Moderate (tank setup)	Low (fixed facility)
Specimen handling	Minimal (dry, brief exposure)	Submerged (wet)	Destructive (fixation required)
Internal structure	Not captured	Not captured	Fully captured
Surface detail	High (sculpted)	Moderate (photographic)	High (volumetric)
Operator skill	High (software proficiency)	Low–Moderate	Low
Best application	External morphology, education	Rapid field documentation	Internal anatomy, dense structures

### 4.4 Applications and Future Directions

#### 4.4.1 Ecological and Evolutionary Research

The 3D modeling workflow supports quantitative morphological analyses that have previously been difficult to scale. Roscian et al. (2022) identified habitat‐related beak shape differences (pelagic vs. benthic species) using 209 specimens; our method could facilitate comparable large‐scale analyses, pending further validation. The ability to rapidly prototype physical models also enables biomechanical testing of form–function relationships (Uyeno and Clark 2015).

For population ecology, accurate 3D models from large numbers of beak specimens have direct implications for demographic inference. Beak morphometrics are routinely used to estimate age and growth in cephalopod populations (Perales‐Raya et al. 2010; Liu et al. 2020), and 3D shape data may improve the precision of such estimates by capturing allometric relationships invisible to 2D caliper measurements. In trophic ecology, quantitative 3D models can refine diet reconstruction from stomach‐content analysis, as subtle shape differences among congeneric prey species are often better resolved in three dimensions than from linear measurements alone. Validated 3D models reduce inter‐operator measurement bias and improve reproducibility across studies, which is critical for meta‐analyses of population structure and geographic variation. The low cost and portability of the prism‐assisted photogrammetry system allow field researchers to generate 3D morphological data in remote or resource‐limited settings, extending the spatial and temporal scope of beak‐based ecological monitoring.

#### 4.4.2 Science Communication and Education

Physical 3D‐printed models address a practical limitation in cephalopod research: the restricted availability of beak specimens due to their small size, fragility, and preservation requirements. Museum exhibitions, classroom instruction, and public outreach can use these replicas to illustrate feeding biomechanics, species diversity, and evolutionary adaptation. Digital files (provided as Data S1) allow wider distribution without physical specimen transport.

#### 4.4.3 Methodological Extensions

Several technical improvements could enhance the workflow:

1. Automation: Python scripting in ZBrush could automate measurement extraction, reducing operator time and error.
2. Material simulation: Subsurface scattering shaders (Substance Designer, V‐Ray) could better replicate transparent edge regions.
3. Multi‐scale integration: Combining surface models with micro‐CT internal data would yield more complete anatomical representations.
4. Machine learning: Training convolutional neural networks on reference image libraries could enable automated contour extraction and direct 3D reconstruction.

### 4.5 Limitations

Several constraints should be acknowledged:

1. Thickness limitation: The 2 mm minimum print thickness requirement prevents accurate replication of the thinnest beak edges (~0.1 mm). Transparent resins with post‐processing painting could partially address this.
2. Operator dependence: Digital sculpting quality depends on operator skill and anatomical knowledge. Standardized training protocols and quality control metrics are needed.
3. Internal structure: Our method captures external morphology only; internal features (e.g., growth rings) require micro‐CT or histological sectioning.
4. Pigmentation: Current printing materials cannot replicate the natural transparency gradient; hand‐painting is required for visual accuracy.
5. Sample size: The limited sample size precluded large‐scale morphological comparisons within or between species. Future studies should expand sample sizes across growth stages and species to validate method stability in large‐scale analyses.

## 5 Conclusions

We describe a validated, cost‐effective workflow for rapid 3D reconstruction of small, delicate biological specimens, using cephalopod beaks as a case study. The combination of prism‐assisted photogrammetry, digital sculpting, and micro‐CT validation yielded linear and angular accuracies (< 2% and < 3°, respectively) comparable to more expensive methods, while reducing equipment costs and acquisition time. This approach removes a technical bottleneck that has limited the scaling of beak‐based ecological analyses to large sample sizes, with direct implications for population structure inference, diet reconstruction from stomach contents, and demographic modeling. The use of accessible software and hardware lowers the barriers to 3D morphological documentation and may facilitate wider adoption in ecology, biodiversity research, and science education.

## Author Contributions

**Lele Xu:** writing – original draft (lead), writing – review and editing (lead). **Yongqin Li:** validation (equal), visualization (lead). **Jiechun Chen:** investigation (supporting), methodology (equal). **Dongmei Huang:** investigation (supporting), methodology (supporting). **Yao Liu:** methodology (equal), project administration (equal). **Liyun Wang:** project administration (lead). **Bilin Liu:** funding acquisition (equal), project administration (equal).

## Funding

This work was supported by Zhanjiang Science and Technology Bureau (2023A01020, 2023A01024), Lingnan Normal University (000302001175), National Key R&D Program of China (2023YFD2401302), Shanghai Institutions of Higher Learning (GZ2022011).

## Ethics Statement

No live animals were used in this study. Specimens were obtained post‐mortem from commercial fisheries, eliminating the need for ethical approval.

## Conflicts of Interest

The authors declare no conflicts of interest.

## Supporting information


**Data S1:** ece373992‐sup‐0001‐Supinfo.zip.

## Data Availability

The data that support the findings of this study are available within the article and its Supporting Information. Source data are provided with this paper.
